# Post-translational regulation of metabolic checkpoints in plant tetrapyrrole biosynthesis

**DOI:** 10.1093/jxb/erac203

**Published:** 2022-05-10

**Authors:** Peng Wang, Shuiling Ji, Bernhard Grimm

**Affiliations:** Institute of Biology/Plant Physiology, Humboldt-Universität zu Berlin, Philippstraße 13 (Haus 12), 10115 Berlin, Germany; Institute of Biology/Plant Physiology, Humboldt-Universität zu Berlin, Philippstraße 13 (Haus 12), 10115 Berlin, Germany; Institute of Biology/Plant Physiology, Humboldt-Universität zu Berlin, Philippstraße 13 (Haus 12), 10115 Berlin, Germany; University of Illinois, USA

**Keywords:** Aminolevulinic acid, chlorophyll, chloroplast biogenesis, metabolic control, pigment synthesis, post-translational control, protein–protein interaction, subcellular compartments, tetrapyrrole biosynthesis

## Abstract

Tetrapyrrole biosynthesis produces metabolites that are essential for critical reactions in photosynthetic organisms, including chlorophylls, heme, siroheme, phytochromobilins, and their derivatives. Due to the paramount importance of tetrapyrroles, a better understanding of the complex regulation of tetrapyrrole biosynthesis promises to improve plant productivity in the context of global climate change. Tetrapyrrole biosynthesis is known to be controlled at multiple levels—transcriptional, translational and post-translational. This review addresses recent advances in our knowledge of the post-translational regulation of tetrapyrrole biosynthesis and summarizes the regulatory functions of the various auxiliary factors involved. Intriguingly, the post-translational network features three prominent metabolic checkpoints, located at the steps of (i) 5-aminolevulinic acid synthesis (the rate-limiting step in the pathway), (ii) the branchpoint between chlorophyll and heme synthesis, and (iii) the light-dependent enzyme protochlorophyllide oxidoreductase. The regulation of protein stability, enzymatic activity, and the spatial organization of the committed enzymes in these three steps ensures the appropriate flow of metabolites through the tetrapyrrole biosynthesis pathway during photoperiodic growth. In addition, we offer perspectives on currently open questions for future research on tetrapyrrole biosynthesis.

## Teaser

The recently described regulatory factors and their functions at three main checkpoints of tetrapyrrole biosynthesis, which impact stability, enzymatic activity, and spatial organization of the committed enzymes of these three steps of the pathway, are reviewed.

## Introduction

**Fig. 1.**
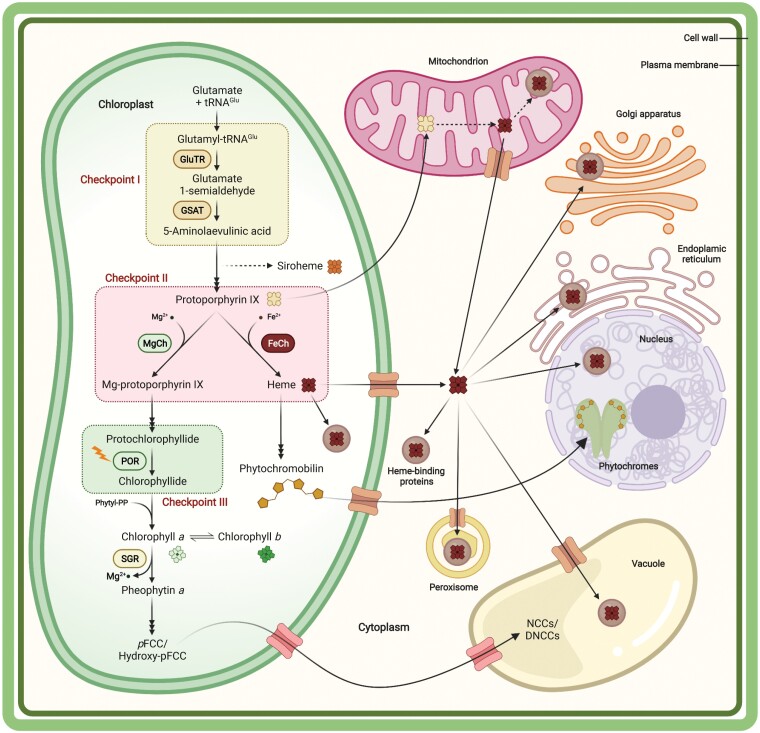
Checkpoints for post-translational control of tetrapyrrole biosynthesis and distribution in plant cells. Tetrapyrroles, including chlorophylls, heme, siroheme, and phytochromobilin, are synthesized in plastids via the tetrapyrrole biosynthesis pathway. Here we emphasize three metabolic checkpoints, which are tightly regulated at the post-translational level. The first checkpoint controls the first committed and rate-limiting step in the pathway, the synthesis of 5-aminolevulinic acid, which is catalyzed by glutamyl-tRNA reductase (GluTR) and glutamate 1-semialdehyde aminotransferase (GSAT). The second checkpoint is located at the point at which protoporphyrin IX is directed into the chlorophyll and heme synthesis branches of the pathway. Magnesium chelatase (MgCh) and ferrochelatase (FeCh) insert magnesium and iron ions into protoporphyrin IX to produce Mg-protoporphyrin IX and heme, respectively. The third checkpoint is the regulation of the light-dependent protochlorophyllide oxidoreductase (POR), which catalyzes the reduction of protochlorophyllide *a* to chlorophyllide *a*. Chlorophyllide a is esterified with phytyl diphosphate (Phytyl-PP) to produce chlorophyll *a*, which can be oxidized to form chlorophyll *b*. Magnesium dechelatase, also called STAY-GRREN (SGR), removes magnesium from chlorophyll *a* and initiates chlorophyll degradation. Tetrapyrroles are widely distributed in various compartments of plant cells. Chlorophylls interact with various chlorophyll-binding proteins to form the two photosystems and their respective light-harvesting complexes in chloroplasts, but their catabolic intermediates—primary fluorescent chlorophyll catabolites (pFCCs) and C32-hydroxylated pFCCs (hydroxy-pFCCs)—are exported from plastids and finally converted into non-fluorescent chlorophyll catabolites (NCCs) and formyloxobilin-type NCCs (DNCCs) in the vacuole. Heme is associated with various heme-binding proteins, which are found in all cellular compartments. It is still unclear whether mitochondria can use protoporphyrin IX derived from plastids to produce heme, which is further distributed to other cellular compartments. Phytochromobilin is incorporated into phytochromes, which are translocated into the nucleus. The membrane transporters involved in tetrapyrrole trafficking through membrane systems are largely unknown.

Macrocyclic tetrapyrroles are vital constituents of many essential pathways in all organisms, regardless of whether they are derived from metabolic precursors or acquired as end-products from other sources, such as symbionts (Kořený *et al.*, 2013; Richtova *et al*., 2021). In plants, multiple end-products of the tetrapyrrole biosynthesis (TBS) pathway (Fig. 1) serve different purposes during development. Chlorophyll (Chl) is essential for photosynthesis, as it absorbs light to fuel photosynthetic electron transfer across the thylakoid membranes of the chloroplasts. Heme is a cofactor in many redox reactions, binds gases, such as O_2_, CO, and NO, and acts as a signaling component in intracellular communication. Phytochromobilin provides the chromophore for the photoreceptor phytochrome during photomorphogenesis. In addition, other bilins are used for light harvesting in cyanobacteria and red algae, and serve as potential signaling components in green and red algae, as well as in both photosynthetic and non-photosynthetic bacteria. Siroheme functions as an essential cofactor in the assimilation of nitrogen and sulfur mediated by nitrite and sulfite reductases.

The TBS pathway consists of up to 20 highly regulated enzymatic steps (Brzezowski *et al.*, 2015; Herbst *et al.*, 2019) and begins as a linear reaction sequence before branching out to form various classes of end-products. Thus, TBS can be divided into the following subsections: (i) 5-aminolevulinic acid (ALA) synthesis, (ii) porphyrin synthesis, (iii) siroheme synthesis, (iv) Chl synthesis, (v) heme synthesis, (vi) Chl recycling, and (vii) the Chl cycle (Wang and Grimm, 2021). The last subsections two mediate the final steps of Chl synthesis and the initial steps of Chl catabolism. Many tetrapyrroles are photoreactive or readily oxidized, and this can promote the generation of reactive oxygen species and oxidatively degrade lipids. Therefore, TBS must be tightly controlled, and the pathway as a whole is organized into multi-enzyme complexes located at defined subcompartmental sites within the plastids (Sobotka *et al.*, 2014; Wang and Grimm, 2015). It is likely that each individual enzymatic step is extensively regulated by a wide range of processes that fine-tune the catalytic activity and stability of the relevant enzymes (Herbst *et al.*, 2019), but specific enzymes catalyze the crucial rate-determining steps in the metabolic pathway.

**Fig. 2.**
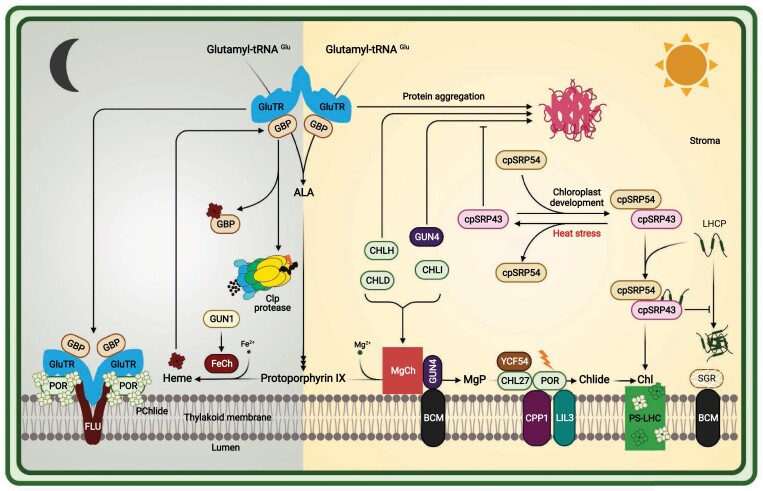
Summary of post-translational control mechanisms in tetrapyrrole biosynthesis. Tetrapyrrole biosynthesis is active in the light when the rate-limiting enzyme, glutamyl-tRNA reductase (GluTR), forms a soluble Y-shaped dimer and interacts with GluTR-binding protein (GBP). In the dark, the activity of GluTR is inhibited by the incorporation of a FLUORESCENT (FLU) dimer to form a membrane-bound GBP–GluTR–FLU– protochlorophyllide oxidoreductase (POR)–protochlorophyllide (PChlide) inactivation complex. It has been reported that FLU also inhibits GluTR under fluctuating light conditions. GENOMES UNCOUPLED 1 (GUN1) can stimulate ferrochelatase I (FeCh) to produce heme. Heme synthesized in the chloroplast can interact with GBP, and heme-bound GBP is released from GluTR. Free soluble GluTR is accessible to proteolysis by Clp proteases. Magnesium chelatase (MgCh) comprises the catalytic subunit CHLH and two AAA^+^ protein subunits, CHLD and CHLI. The MgCh complex is formed by the interaction of CHLH with the two hexameric CHLD and CHLI rings. GENOMES UNCOUPLED 4 (GUN4) binds protoporphyrin and Mg protoporphyrin, and stimulates MgCh activity by interacting with CHLH. The two transmembrane BALANCE OF CHLOROPHYLL METABOLISM (BCM1/2) proteins optimize chlorophyll (Chl) biosynthesis by stimulating MgCh activity via interaction with GUN4, and by attenuating Chl breakdown by inducing the degradation of the Mg dechelatase SGR. YCF54 acts as a positive regulator of Mg-protoporphyrin monomethyl ester cyclase (CHL27). The J-domain-containing CHAPERONE-LIKE PROTEIN FOR POR1 (CPP1) and light-harvesting-like 3 (LIL3) are essential for the stability of POR. Newly synthesized Chls are incorporated into various Chl-binding proteins, such as light-harvesting Chl-binding proteins (LHCPs), to form the photosystem–light-harvesting antenna (PS-LHC) complexes embedded in the thylakoid membranes. Chloroplast signal recognition particle 43 (cpSRP43) is involved in post-translational transfer of LHCPs from the stroma into the thylakoid membranes. Then, cpSRP43 interacts with unfolded LHCPs and cpSRP54 to form a transit complex, which efficiently prevents the aggregation of hydrophobic LHCPs in the aqueous stroma. Apart from the efficient prevention of LHCP aggregation in the aqueous stromal compartment, the ATP-independent molecular chaperone activity of cpSRP43 also protects GluTR, CHLH, and GUN4 from aggregation during chloroplast development and heat stress. Heat-induced dissociation of cpSRP43 from cpSRP54 enables cpSRP43 to chaperone these three tetrapyrrole biosynthetic enzymes under heat stress.

Our intention in this review is to provide a description of the known post-translational mechanisms involved in plant TBS, highlighting recent findings on control strategies and novel participating actors (Fig. 2). So far, three enzymes—glutamyl-tRNA reductase (GluTR), Mg chelatase (MgCh), and NADPH-dependent protochlorophyllide oxidoreductase (POR)—have attracted particular attention, as they have been shown to associate with many regulatory factors. GluTR catalyzes the first committed step in ALA synthesis, MgCh functions at the beginning of Chl synthesis, and POR mediates the NADPH- and light-dependent reduction of protochlorophyllide (PChlide) to chlorophyllide (Chlide). Given their strategic positions within the metabolic pathway, we believe that these three enzymatic steps are the most relevant for the overall control of metabolic flows in Chl synthesis. They represent crucial metabolic checkpoints of TBS in plants (Fig. 1). GluTR acts as the rate-limiting enzyme in TBS, and its activity and stability largely determine the available supply of ALA, the first specific precursor of tetrapyrroles. In photosynthetically active plant organs, MgCh acts at the decisive branch point of porphyrin synthesis, where protoporphyrin IX is predominantly allocated to the Mg branch for Chl synthesis. In angiosperms, the light-dependent activity of POR restricts Chl synthesis to the light period. This enzyme activity must therefore have evolved in tandem with the tightly controlled suppression of ALA synthesis to prevent the simultaneous accumulation of photoreactive tetrapyrrole metabolites.

We discuss the recently described regulatory components and their functions at each of the three checkpoints in the following sections. The current state of exploration reveals that, while many such factors are restricted to a single checkpoint, others interact with several TBS enzymes. For further information on tetrapyrroles in unicellular photoautotrophs, including algae and oxygenic and anaerobic bacteria, we refer readers to the recent reviews by Dailey *et al.* (2017) and Bryant *et al.* (2020). For the more specific aspects of tetrapyrrole-derived retrograde signaling, see Shimizu and Masuda (2021), and for details of the coordination of TBS with the synthesis of Chl-binding proteins, see Wang and Grimm (2021).

## Checkpoint I: post-translational control of ALA synthesis

In plants, two enzymes convert activated glutamate into ALA: GluTR and glutamate-1-semialdehyde aminotransferase (GSAT). The glutamate moiety of glutamyl-tRNA^Glu^, which is the substrate for plastidial translation and TBS, is transferred to the thiol group of a conserved cysteine in the catalytic center of GluTR, and the thioester bond is then reduced to an aldehyde by NADPH (Moser *et al.*, 1999). The resulting GSA is transaminated to ALA by GSAT, which mediates the transfer of an amino group from C2 to C1. As indicated above, ALA synthesis is the rate-limiting step for the entire pathway, and recent studies have revealed that GluTR is crucial for the precise control of the amount of ALA made available during photoperiodic growth. Several factors that interact with GluTR have been identified and characterized, and, thus, underline the importance of the post-translational control of GluTR (Fig. 2).

### *FLUORESCENT* gene

The *FLUORESCENT* (*FLU*) gene was identified in a mutant screen for deregulated TBS in etiolated seedlings (Meskauskiene *et al.*, 2001). Mutants that exhibited a cell-death phenotype when seedlings were exposed to light were selected. The necrotic seedlings accumulated excess PChlide, indicating that ALA synthesis was not adequately repressed during incubation of the *flu* seedlings in darkness (Meskauskiene *et al.*, 2001). Upon light absorbance by the accumulated free PChlide, excessive amounts of singlet oxygen are generated, which ultimately causes extensive photo-oxidative damage and cell death via the action of EXECUTER proteins (Wagner *et al.*, 2004). Only under continuous illumination do young *flu* seedlings develop into normal green seedlings. However, subsequent dark incubation of *flu* mutants leads to a necrotic cell-death phenotype in leaves when the seedlings are reilluminated. The FLU protein is a membrane-bound member of the tetratricopeptide-repeat (TPR) family in plants (Bohne *et al.*, 2016). FLU possesses three 34-amino-acid-long TPR motifs, which are assumed to serve as binding surfaces for various proteins, and FLU interacts with both GluTR and POR (Meskauskiene and Apel, 2002; Kauss *et al.*, 2012). It has been proposed that accumulating PChlide bound to POR ­triggers the ­inactivation of GluTR by interacting with FLU (Richter *et al.*, 2010). While this suggestion is now generally accepted, the precise molecular mechanism of FLU-mediated inactivation of GluTR is still unclear, and several questions remain unanswered. How is FLU stimulated to bind to GluTR, which was initially described as a soluble protein in the stroma (Pontoppidan and Kannangara, 1994)? How does the membrane-bound FLU attract stroma-localized GluTR to the membrane? How is GluTR activity modulated under different light intensities and stress conditions?

The suppression of ALA synthesis in darkness is a consequence of the light dependence of POR function. However, FLU is also required for the fine-tuning of ALA synthesis during light exposure. Recent studies have shown that FLU action is also necessary for the adjustment of the rate of ALA synthesis in fluctuating light conditions (Hou *et al.*, 2019). Thus, the *flu* mutant accumulates elevated levels of PChlide relative to the wild type under varying light intensities, due to inadequate control of ALA synthesis. FLU-overproducing seedlings show a pale green phenotype (Hou *et al.*, 2019). This is a reflection of excessive FLU-mediated repression of ALA synthesis upon light exposure, as more GluTR accumulates at the membrane than in the wild type. Conversely, in *flu* mutants a larger fraction of GluTR is found in the stroma than in wild-type and FLU-overproducing lines (Hou *et al.*, 2019). However, assuming that FLU contributes to the control of ALA synthesis in the light, how does it sense changes in light intensity? Obviously, the saturating amounts of PChlide responsible for dark suppression of ALA synthesis at the level of POR cannot serve as the signal under varying light conditions. Hence, the nature of the (presumably more specialized) control mechanism awaits elucidation.

By Blue Native gel electrophoresis and co-immunoprecipitation, FLU was found to assemble into a large protein complex made up of POR and other proteins of late Chl biosynthesis, including the Mg protoporphyrin monomethylester cyclase subunit CHL27, geranylgeranyl reductase (GGR, also called CHLP), and divinyl reductase (DVR) (Kauss *et al.*, 2012). In the dark-incubated samples, these authors also identified GluTR as part of the FLU-containing inactivation complex. However, it cannot be excluded that additional factors are involved in the inactivation of GluTR by FLU in the dark and/or in the light.

### GluTR-binding protein and heme-dependent GluTR degradation

Efforts to characterize proteins that interact with GluTR by the yeast two-hybrid approach resulted in the identification of the GluTR-binding protein (GBP). GBP tightly binds to the N-terminal end of GluTR. This stretch of the first N-terminal 30 amino acid residues of mature GluTR was initially designated as a heme-binding domain (HBD), the deletion of which diminished the feedback inhibition of GluTR activity by heme (Vothknecht *et al.*, 1998). Almost 20 years later, removal of the HBD was shown to prevent the interaction of GluTR with GBP (Apitz *et al.*, 2016). The bimolecular interaction was confirmed by a crystal structure of dimeric, Y-shaped Arabidopsis GluTR combined with dimeric GBP and, subsequently, by a crystallized heterotrimeric complex consisting of GluTR, GBP, and the TPR domain of FLU (Zhao *et al.*, 2014; Fang *et al.*, 2016). In comparison to the wild type, the *gbp* knockout mutant did not show any remarkable phenotypic changes during growth in a greenhouse or in growth chambers (Czarnecki *et al.*, 2011). An allelic *proton gradient regulation 7* (*pgr7*) mutant was independently identified in a screen for low non-photochemical quenching of Chl fluorescence (Jung *et al.*, 2010). Due to the observed impairment of efficient photosynthetic electron transport in this mutant, a contribution of PGR7 to the assembly of the cytochrome b6f complex has been suggested (Jung *et al.*, 2010).

Zhao *et al.* (2014) noted the structural similarity between the N- and C-terminal domains of GBP and the corresponding domains of HugZ (heme utilization gene Z; Hu *et al.*, 2011), and pointed to a potential role of GBP in heme binding. More recently, biochemical studies have revealed heme binding by GBP, and a post-translational feedback effect of heme on ALA synthesis. It was demonstrated that an elevated heme content (which was achieved by feeding dark-incubated leaves with ALA) affects the stability of GluTR. These *in vivo* studies revealed that excess heme binds to GBP and weakens the contact between GBP and GluTR. The concurrent loosening of the tight bonds between GBP and GluTR makes GluTR more accessible to the selector and chaperone subunits of the caseinolytic protease (Clp) and, in consequence, more susceptible to proteolytic degradation. The lower GluTR content leads to a reduction in the rate of ALA synthesis. As a result, the GluTR content is diminished, while free heme accumulates in chloroplasts (Richter *et al.*, 2019). Thus, GBP serves as a heme-binding protein for the heme-dependent feedback control of GluTR stability. As one of the end-products of the pathway, heme has long been proposed as a regulator of plant TBS (Cornah *et al.*, 2003), albeit without any substantial indications as to how heme might be involved in feedback control. Since the control of heme synthesis should be counteracted immediately after the accumulation of free heme, the modulation of ALA synthesis is an appropriate post-translational target for this regulatory function of heme. Interestingly in this context, an *fc2* (knockout mutation of *ferrochelatase*2) mutant did not show heme-dependent GluTR degradation, while the *fc1* knockdown mutant (knockout mutations of *ferrochelatase* 1 are lethal) was not impaired in GluTR degradation (Richter *et al.*, 2019).

### GluTR degradation by Clp proteases

As mentioned above, heme-based regulation of ALA synthesis does not lead to GluTR inactivation but to its proteolytic degradation. The N-terminal domain of GluTR was also shown to interact with the Clp subunits ClpC1, ClpF, and ClpS (Nishimura *et al.*, 2015; Apitz *et al.*, 2016). The interdependency of GBP and Clp subunits on GluTR was confirmed when the heme-mediated feedback control of ALA synthesis was first reported. When heme binding to GBP reduces its affinity for GluTR, the Clp subunits can target GluTR for proteolysis. Consequently, Richter *et al.* (2019) suggested that the N-terminal segment of GluTR should be designated as a regulatory domain rather than an HBD, since GluTR does not bind heme and the region is a site for interaction with several proteins (see also the chaperone function of cpSRP43, discussed below).

Thus, as indicated by the mode of action of FLU, GBP, and the Clp protease, quite distinct regulatory mechanisms affect the rate-limiting function of GluTR. FLU inactivates GluTR, GBP stabilizes GluTR, and heme triggers Clp-dependent GluTR proteolysis. Apart from some general observations of reduced GluTR contents in single and double mutants for thioredoxin (TRX) isoforms and NADPH-dependent ­thioredoxin reductase C (NTRC), no stimulatory effects of other factors on GluTR and ALA synthesis have been described so far. Stimulation of ALA synthesis is primarily based on transcriptional control of the GluTR-encoding *HEMA* gene (Kobayashi and Masuda, 2016). In this context, it is significant that ALA synthesis is correlated with the amount of soluble GluTR, while membrane-bound GluTR reflects reduced rates of ALA synthesis (Schmied *et al.*, 2018; see also the last section of this review).

## Checkpoint II: auxiliary factors involved in the insertion of Mg into protoporphyrin IX

MgCh executes the first committed step in the Chl synthesis pathway, the incorporation of Mg^2+^ into protoporphyrin IX. MgCh is a multisubunit enzyme comprising a catalytic CHLH subunit and two AAA^+^ subunits (CHLD and CHLI), which dynamically assemble and disassemble for the enzymatic reaction (Masuda, 2008). It has been proposed that CHLD and CHLI assemble into two layered hexameric rings, which interact with CHLH (Axelsson *et al.*, 2006). ATP hydrolysis catalyzed by CHLI drives the synthesis of Mg protoporphyrin and the disassembly of the MgCh complex.

MgCh is a major target of post-translational control mechanisms. *GENOMES UNCOUPLED 4* (*GUN4*) was initially identified in the *gun* mutant screen (Susek *et al.*, 1993; Mochizuki *et al.*, 2001). Apart from its contribution to retrograde signaling, GUN4 protein binds protoporphyrin and Mg protoporphyrin, and stimulates Mg chelation by interacting with CHLH (Larkin *et al.*, 2003). Although GUN4 is a soluble protein, it was reported that after porphyrin binding GUN4 displayed a stable association with the thylakoid membranes to optimize the interaction of GUN4 with CHLH (Adhikari *et al*., 2009, 2011). The membrane binding of GUN4 seems to be crucial for the initiation of Chl biosynthesis, as the Mg porphyrins become more and more hydrophobic and the enzymes that follow MgCh, such as Mg protoporphyrin methyltransferase (CHLM), Mg protoporphyrin monomethylester cyclase, and protoporphyrinogen oxidase, are membrane associated (Joyard *et al.*, 2009).

Homologs of GUN4 are widely distributed among photosynthetic organisms. Compared with its counterparts in cyanobacteria and green algae, GUN4 in land plants has a C-terminal extension, which is phosphorylatable (at Ser264 of Arabidopsis GUN4) (Richter *et al.*, 2016a). Interestingly, phospho-mimicking versions of GUN4 stimulate the *in vitro* Mg chelation of recombinant MgCh subunits to a lesser extent than wild-type or Ser→Ile substitution mutants of GUN4. Expression of phospho-mimicking GUN4(S264I) in the *gun4* null mutant results in pale green pigmentation (indicating reduced Chl biosynthesis), while wild-type and GUN4(S264I) express similar amounts of Chl (Richter *et al.*, 2016a). Since GUN4 is preferentially phosphorylated in the dark, it is thought that the attenuation of MgCh activity decreases the flux of Mg porphyrins into the dark-inhibited Chl-synthesizing pathway and thus contributes to the strict repression of PChlide reduction by the light-dependent POR (Richter *et al.*, 2016a).

The regulatory effects of plastid-localized TRXs and NTRC on the successive steps in Chl synthesis catalyzed by the MgCh subunit CHLI and the Mg protoporphyrin methyl transferase (CHLM) have been intensively investigated (Ikegami *et al.*, 2007; Richter *et al*., 2013, 2016b). The apparent degradation of these proteins in *trx* and *ntrc* mutants points to a new role of redox control in TBS in comparison to that in the Calvin–Benson cycle, when key enzymes, such as glyceraldehyde 3-phosphate dehydrogenase, fructose-1,6-bisphosphatase and seduheptulose-1,7-bisphosphatase, are activated in a redox-dependent manner. Thus, the current view of redox control in TBS emphasizes redox-dependent protein degradation in mutants that are deficient in m- and f-type TRX variants and/or NTRC (Wittmann *et al.*, 2021).

### The chaperone function of cpSRP43 prevents the aggregation of GluTR, CHLH, and GUN4

The trafficking of light-harvesting Chl *a*/*b*-binding proteins (LHCPs) and the assembly of plastid-encoded proteins of photosystems I and II require the assistance of chloroplast signal recognition particle (cpSRP) components. cpSRP43 and cpSRP54 belong to these pathways and are named based on their respective molecular weights (Li *et al.*, 1995; Schuenemann *et al*, 1998). The two proteins are responsible for the translocation of hydrophobic LHCPs from the translocon in the inner envelope complex (TIC) through the stroma and subsequent docking at the thylakoid membrane. During this process, cpSRP43 binds to unfolded LHCPs and cpSRP54 to form a soluble transit complex, in which LHCPs safely cross the aqueous stroma (Akopian *et al.*, 2013; Ziehe *et al.*, 2018). cpSRP43 acts as an ATP-independent molecular chaperone to protect LHCPs from aggregation (Falk and Sinning, 2010; Jaru-Ampornpan *et al.*, 2010). The interaction of cpSRP54 with the thylakoid-membrane-localized SRP-receptor protein cpFtsY helps to dock the cpSRP/LHCP transit complex to the stromal side of the thylakoid membrane (Jaru-Ampornpan *et al.*, 2007; Akopian *et al.*, 2013). Upon GTP hydrolysis catalyzed by cpSRP54 and cpFtsY, the LHCP is finally released from the transit complex and inserted into the thylakoid membrane with the aid of the insertase ALBINO 3 (Falk *et al.*, 2010; Horn *et al.*, 2015).

LHCPs bind Chl *a*/*b* and carotenoids, which are responsible for light absorption and energy transfer from the peripheral antenna complexes of photosystems I and II (Allen *et al.*, 2011; Jarvis and Lopez-Juez, 2013). The dynamically changing size of the antenna complexes in response to varying light intensities and other fluctuating environmental factors requires precise coordination of LHCP biogenesis with Chl biosynthesis. Recent studies have pinpointed new targets of the chaperone function of cpSRP43, which are implicated in the checkpoints of ALA synthesis and Mg chelation. The reduced stability of GluTR, CHLH, and GUN4 in the *cpsrp43* knockout mutant and the interaction of cpSRP43 with these three TBS proteins have revealed them as new clients of cpSRP43 (Wang *et al.*, 2018; Ji *et al.*, 2021). cpSRP43 binds to the N-terminus of GluTR, which includes two aggregation-prone regions (APRs). It was proposed that APR motifs could cause aggregation of the GluTR nascent chain, and that cpSRP43 chaperones GluTR and prevents its aggregation, most likely by occluding the APRs and facilitating correct folding through its intrinsic chaperone activity (Wang *et al.*, 2018). Since CHLH and GUN4 are essential for MgCh activity, cpSRP43 may also stabilize MgCh to maintain Chl biosynthesis. The chaperone function of cpSRP43 is suggested to connect LHCP translocation with Chl biosynthesis by safeguarding the homeostasis of highly regulated proteins of TBS (Wang *et al.*, 2018; Ji *et al.*, 2021).

cpSRP43 consists of an N-terminal chromodomain 1 (CD1), four ankyrin repeats (Ank 1–4), and two additional chromodomains (CD2 and CD3) at the C-terminus (Klimyuk *et al.*, 1999; Goforth *et al.*, 2004; Stengel *et al.*, 2008). CD1, Ank 1–4, and the linker sequence between Ank4 and CD2 constitute the substrate-binding domain (SBD), which is sufficient to chaperone LHCPs (Liang *et al.*, 2016). In contrast, the SBD and CD2 are required for the chaperone activity of cpSRP43 towards GluTR, CHLH, and GUN4 (Ji *et al.*, 2021). Since the CD2 domain also binds to cpSRP54 (Kathir *et al.*, 2008), the interaction of both cpSRP components enhances the chaperone activity for LHCPs (Liang *et al.*, 2016), but suppresses the chaperoning of TBS proteins by cpSRP43 (Ji *et al.*, 2021). However, increased temperature attenuates the cpSRP43–cpSRP54 interaction, while the release of cpSRP43 enables it to bind to GluTR, CHLH, and GUN4 (Ji *et al.*, 2021). In summary, apart from LHCP trafficking in the chloroplast, cpSRP43 functions in the chaperoning of GluTR, CHLH, and GUN4. This activity of cpSRP43 is promoted under heat-shock conditions. It has been suggested that the action of cpSRP43 ensures a sufficient supply of Chl for Chl-binding proteins, including LHCPs, during fluctuating environmental conditions (Wang *et al.*, 2018; Ji *et al.*, 2021).

### BALANCE OF CHLOROPHYLL METABOLISM

After the greening of emerging seedlings, a stable level of Chl is maintained to ensure adequate photosynthesis, before Chl is drastically degraded during plant senescence. Given that levels of Chl are determined by the relative rates of Chl biosynthesis and breakdown, plants possess a regulatory mechanism that controls the balance between these two processes in the course of plant development. It was recently shown that two BALANCE OF CHLOROPHYLL METABOLISM (BCM) isoforms (designated BCM1 and BCM2) serve as post-translational factors that stimulate Chl biosynthesis and ­simultaneously attenuate Chl breakdown in Arabidopsis (Wang *et al.*, 2020). The two *BCM* genes code for highly conserved protein sequences, but display differential expression patterns during leaf development. The expression of *BCM1* is highly active in young green leaf tissues, but declines upon the onset of leaf senescence, whereas the *BCM2* gene is predominantly expressed in senescent leaves. Concurrent mutation of both BCM isoforms in Arabidopsis leads to a dual phenotype characterized by abnormally pale leaves in young seedlings and premature leaf senescence in mature plants (Wang *et al.*, 2020).

Protein–protein interaction analysis and an *in vitro* enzyme assay suggested that BCMs stimulate MgCh activity via interaction with GUN4 (Wang *et al.*, 2020). In addition, BCMs are able to interact with the magnesium dechelatase isoform SGR1 (the so-called STAY-GREEN 1), which is the predominant isoform among the three Arabidopsis Mg dechelatase variants that remove the central magnesium ion from Chl *a* to form pheophytin *a* (Shimoda *et al.*, 2016). The interaction of BCMs with SGR1 leads to the destabilization of SGR1 (Wang *et al.*, 2020). All in all, it is suggested that the two BCMs primarily regulate Chl homeostasis by stimulating MgCh activity and by destabilizing magnesium dechelatase.

BCM paralogs are highly conserved in land plants, but are not found in other photosynthetic organisms such as cyanobacteria and green algae. It has been shown that the mutation of a BCM paralog severely affects the green pigmentation of soybean leaves (Liu *et al.*, 2020; Yamatani *et al.*, 2021), soybean seed coat (Wang *et al.*, 2018), and tomato fruits (Liu *et al.*, 2021). Future studies will undoubtedly yield more insight into the molecular mechanisms of BCM action in the regulation of Chl metabolism and other aspects of chloroplast development.

### GENOMES UNCOUPLED 1 and its interaction with TBS enzymes

GENOMES UNCOUPLED 1 (GUN1) is a plastid-localized factor that interacts with several TBS enzymes. The *GUN1* gene was one of five *GUN* loci identified in a mutant screen for impaired plastid-derived retrograde signaling that modifies nuclear gene expression (Koussevitzky *et al.*, 2007). *GUN1* codes for a pentatricopeptide repeat protein, while the other *gun* mutants (*gun2–gun5*) each bear point mutations in genes involved in tetrapyrrole metabolism (Susek *et al.*, 1993; Mochizuki *et al.*, 2001; Larkin *et al.*, 2003; Strand *et al.*, 2003). All the *gun* mutants share a common phenotype, namely significantly reduced repression of transcript levels of photosynthesis-associated nuclear genes (*PhANGs*) when chloroplast biogenesis is impaired, for example, by norflurazon or lincomycin treatment. Essentially all of the published studies on *gun* mutants revealed that *GUN1*-mediated retrograde signaling differs from the pathway affected by *gun2–gun5* (Wu and Bock, 2021).

The minor reduction of leaf pigmentation seen in *gun1* mutants did not immediately suggest a significant ­impairment of TBS. Several attempts were made to unravel the role of GUN1 during plastid biogenesis and plastid-derived retrograde signaling. Diverse plastid-localized processes have been attributed to the interaction of GUN1 with various protein partners, which suggests that it plays a highly complex role. The *gun1* mutation affects plastid gene expression (Koussevitzky *et al.*, 2007), RNA editing, and plastid ribosome assembly. An additional function was proposed for GUN1 in the transcriptional response to perturbed plastid homeostasis, which itself depends on the nucleus-encoded RNA polymerase (NEP) (Tadini *et al.*, 2016). The general maintenance of protein homeostasis in the chloroplasts of young seedlings came into focus when defects in the uptake of nucleus-encoded, plastid-localized proteins were linked to the loss of GUN1 function (Tadini *et al.*, 2016; Wu *et al.*, 2019). Precursor proteins were found to accumulate in the cytoplasm of *gun1* plants as a result of the destabilization of the translocon at the outer envelope of the chloroplast (TOC) and reduced accumulation of TIC100, a component of the translocon at the inner envelope of the chloroplast (TIC) (Tadini *et al.*, 2016). These observations suggested that GUN1 is involved in determining the chloroplast’s capacity for protein import (Wu *et al.*, 2019).

Along the same lines, GUN1 was shown to interact with many different TBS enzymes, including GluTR, GSAT, hydroxymethylbilane synthase, uroporphyrinogen decarboxylase, the MgCh subunit CHLD, and the ferrochelatase isoform FC1 (Tadini *et al.*, 2016; Wu *et al.*, 2019). These interactions of GUN1 with nucleus-encoded TBS enzymes pointed to a potential role for GUN1 in the control of protein translocation into chloroplasts and protein homeostasis within the organelles. Furthermore, GUN1 was reported to bind to heme and have a positive effect on FC1 activity (Shimizu *et al.*, 2019). This is interesting, as it provides a rationale and a mechanism for one specific regulatory role of GUN1 in heme synthesis, which relates to the activity of the two ferrochelatase isoforms FC1 and FC2. Due to their differential expression profiles, it has been suggested that FC1 and FC2 supply heme for different heme-dependent proteins. If GUN1 enhances FC1 activity only, it would affect only the FC1-related heme pool. In this context it is worth mentioning that a subsequently identified *gun6* mutation is characterized by overexpression of *FC1* and defective repression of *PhANG* expression after plastid damage (Woodson *et al.*, 2011). It was proposed that FC1 is responsible for the supply of heme that enhances *PhANG* expression. This hypothesis would imply a specific role for FC1 in the production of heme for extraplastidial heme-binding proteins. However, the observation of derepressed *PhANG* transcription in *gun1* mutants in parallel with the suppression of heme synthesis argues against the idea that GUN1-stimulated FC1 activity provides a positive heme signal for *PhANG* expression.

As mentioned above, these analyses indicate a direct and simultaneous involvement of GUN1 in many aspects of plastidial protein homeostasis, but we are aware that this conclusion gives no indication as to the precise molecular mechanism of GUN1’s action in chloroplast biogenesis during very early seedling development.

## Checkpoint III: the light-dependent protochlorophyllide oxidoreductase

Angiosperms exclusively use the light-dependent POR for the reduction of PChlide (Reinbothe *et al.*, 2010; Heyes *et al.*, 2021). The ternary complex consisting of NADPH, PChlide, and POR is attached to the plastidial membranes. POR is undoubtedly one of the most intensively studied enzymes in Chl synthesis owing to its peculiar catalytic and spectral properties (for reviews, see Gabruk and Mysliwa-Kurdziel, 2015; Heyes *et al.*, 2021) and its role as the target of a complex regulatory mechanism. Here, we address post-translational processes only, and do not discuss the transcriptional control of the *POR* genes.

### POR and FLU-mediated inactivation of GluTR and a complex composed of enzymes of late Chl synthesis

The most important proteins that interact with POR are those involved in sensing its inactivation in the dark, which induces the essential suppression of ALA synthesis. As mentioned earlier in the section on FLU, it is thought that the accumulating PChlide bound to POR leads to the inhibition of ALA synthesis by triggering the binding of FLU to GluTR. The resulting complex is localized at the membrane and, apart from POR, FLU, and GluTR, it includes other enzymes that catalyze late steps of Chl synthesis (Kauss *et al.*, 2012). It has been suggested that this complex is present both in the light and in the dark (Hou *et al.*, 2021). This implies that these proteins are dynamically assembled not only during the repression of ALA synthesis but also during the active flow of metabolites towards Chl. Similarly, OsPORB in rice was shown to interact with OsFLU1 and components of the cyclase reaction, the cyclase homolog YGL8 and the YCF54 homolog OsLCAA, favoring concerted catalysis of these steps in Chl biosynthesis (Kong *et al.*, 2016).

### Light-harvesting-like 3 and YCF54 in POR-containing complexes of the Chl-synthesizing branch of TBS

It was proposed several decades ago that cyclase activity requires membrane and stromal fractions (Walker *et al.*, 1991; Castelfranco *et al.*, 1994). Studies since then have revealed that, in addition to the substrate-binding protein, cyclase function requires both an electron donor and a subunit that links the cyclase with an additional reductase. While ferredoxin has been proposed to donate the electrons for the cyclase reaction (Stuart *et al.*, 2020) with the aid of ferredoxin-NADPH reductase (FNR) (Herbst *et al.*, 2018), YCF54/LCAA (Albus *et al.*, 2012; Hollingshead *et al.*, 2012) has been assigned as an additional scaffold protein to connect the catalytic reaction of the cyclase with a reductase activity. More recent reports suggest that YCF54 also connects the cyclase to POR (Kong *et al.*, 2016).

Light-harvesting-like 3 (LIL3), a member of the light-harvesting complex (LHC) family, interacts with the enzymes POR and GGR/CHLP, which belong to two different pathways, for Chl and terpenoid synthesis, respectively. LIL3 has been proposed to simultaneously stabilize POR and GGR and facilitate the channeling of their respective products, Chlide and geranylgeranyl pyrophosphate or phytyl pyrophosphate, into the subsequent Chl synthase step (Tanaka *et al.*, 2010; Hey *et al.*, 2017). An interaction of LIL3 with Chl synthase has been proposed in barley (Mork-Jansson *et al.*, 2015) but experimentally excluded in Arabidopsis (Hey *et al.*, 2017). Regardless of the resolution of this issue, LIL3-mediated complex formation most probably promotes the channeling of metabolites through these enzymatic steps to ensure the provision of adequate amounts of Chl.

### CHAPERONE-LIKE PROTEIN OF POR1

Apart from its presence in protein complexes that participate in Chl synthesis and the inactivation of ALA synthesis, POR also interacts with another auxiliary factor that helps to preserve its functional structure in a dynamically changing environment. A chaperone function on POR was suggested for the plastid membrane-localized CELL GROWTH DEFECT FACTOR 1 (CDF1) (Kawai-Yamada *et al.*, 2005), which was later renamed CHAPERONE-LIKE PROTEIN OF POR1 (CPP1) (Lee *et al.*, 2013). The knockout of *CDF1/CPP1* in Arabidopsis inhibits embryonic development at the globular stage and prevents the formation of viable seedlings (Kawai-Yamada *et al.*, 2014). CPP1 interacts directly with PORA and PORB. It belongs to the family of DnaJ-like proteins and contains three transmembrane domains. It was suggested that CPP1 can prevent the aggregation of POR, in particular under oxidative stress (Lee *et al.*, 2013; Liu *et al.*, 2016). CPP1 deficiency diminishes POR accumulation and perturbs Chl synthesis, which would also explain the photobleaching and growth-retardation phenotype during illumination, as was initially described for the *cfd1* mutant (Kawai-Yamada *et al.*, 2005). Taken together, these results corroborate a potential function of CDF1/CPP1 as a holdase in the regulation of POR stability.

## Conclusions

Current research on the post-translational control of the TBS pathway has established that the three decisive enzymatic steps—GluTR, MgCh, and POR—are targeted by many regulatory factors. Recent findings have prompted us to focus this review on these three regulatory checkpoints of TBS (Fig. 1). The principal factors involved can be functionally divided into inhibitors (FLU), activators (GUN4), scaffold proteins for stability and complex formation (GBP, BCMs, YCF54, and LIL3), chaperones (cpSRP43 and CPP1), and substrate-binding proteins (GUN4) (Fig. 2). Interestingly, some of these auxiliary factors act on several enzymes simultaneously, and at multiple sites in TBS. It is likely that further factors that contribute to the control and organization of this essential pathway await identification. Moreover, apart from the interdependency of the regulatory factors described here, other control mechanisms intervene at these nodes and at other enzymatic steps of TBS. Examples include thiol-based redox control, phosphorylation, and periodical protein degradation. For these post-translational modulators of TBS, we refer readers to previous reviews (Brzezowski *et al.*, 2015; Wang and Grimm, 2021; Wittmann *et al.*, 2021).

### Open questions and outlook

As always, the discovery of new regulatory processes and the identification of new factors not only expand our knowledge but raise further questions, such as the following.

1. Why have plants invested in the control of essential checkpoints instead of modulating each of the enzymatic steps of TBS? These plant-specific checkpoints of TBS are (i) the rate-limiting step in ALA synthesis, (ii) the point at which protoporphyrin IX is allocated to either the Chl- or the heme-synthesizing branch of the pathway, and (iii) the vital light-dependent enzyme POR (Fig. 1). The three catalytic steps respectively determine the overall rate of production of precursors for the branched pathway, the provision of appropriate amounts of protoporphyrin IX for the Mg and iron branches of TBS (Chl synthesis predominates in leaves, while roots require heme only), and, depending on environmental conditions, the inhibition of the synthesis (or the catalytic conversion) of a TBS intermediate produced by the light-dependent activation of POR. It seems to be absolutely necessary to fine-tune these enzymatic steps. At these checkpoints, control processes define the proper metabolic flux and ensure the supply of needed products in response to developmental, spatial, temporal, and environmental control mechanisms. These checkpoints require multiple control mechanisms, which mediate various activation and inactivation processes. They evolved to match the activity of these highly regulated steps to current needs. The combination of different, often antagonistically and/or synergistically acting mechanisms enables metabolic fluxes to be precisely adjusted, even when a regulatory factor is lacking or disabled.
2. Does light-dependent Chl synthesis provide an evolutionary advantage relative to light-independent Chl synthesis? From our perspective, it might seem more efficient to invest energy in the entire pathway only when its metabolites can be used immediately. In the context of photosynthesis, dark Chl synthesis could be considered to be a risky or wasteful investment. However, light-dependent Chl synthesis requires a degree of forward planning, which is likely to be more demanding, as it requires more complex regulation. The complete inactivation of Chl synthesis in darkness would appear to be a reasonable option only if tetrapyrrole intermediates do not accumulate and the rate-determining step is completely suppressed. However, given the diurnal alternation between light and dark, it makes sense to induce Chl synthesis in preparation for the next daily round of photosynthesis in coordination with the production and assembly of the Chl-containing proteins. Indeed, these processes are transcriptionally induced towards the end of the dark period, before sunrise (Kobayashi and Masuda, 2016).
  It appears that the dependence of Chl synthesis on light at the level of PChlide reduction is beneficial for plants. But the evolution of an exclusively light-dependent mode of Chl synthesis could have occurred only once tight suppression of precursor formation at the level of ALA synthesis became possible. In this context, it is striking that two variants of a FLU-like protein (FLP) are derived from different splice products of the *FLP* gene in the green alga *Chlamydomonas reinhardtii* (Falciatore *et al.*, 2005). FLP interacts with GluTR, and it has been suggested that FLP is required to prevent the overproduction of PChlide and deregulation of the porphyrin pathway during growth in the dark. This may represent an intermediate stage in the repression of ALA synthesis in the dark, although the light-independent POR (DPOR) still converts PChlide to Chlide in *C. reinhardtii*.
3. In particular, the light-dependent and dark-suppressed steps in TBS pose new questions. How is tight control of dark suppression of ALA synthesis achieved? How is FLU triggered to interact with GluTR to inactivate it? It has been proposed that when PChlide has saturated the binding sites on POR, a signal is emitted that activates FLU to interact with GluTR on the membrane. As intensively studied in *flu* mutants, when this mechanism does not work, photoreactive intermediates accumulate. This emphasizes that the benefits of purely light-dependent Chl synthesis are contingent on the availability of reliable regulatory mechanisms.
4. Apart from the essential dark repression of ALA synthesis and the complete blockade of Chl synthesis at the level of PChlide reduction in angiosperms, one must keep in mind that a minor portion of ALA-synthesizing activity is required for the continuous synthesis of heme during the night and the day. Moreover, the quantities required for heme-dependent plastid proteins, or such proteins in other cellular compartments, differ during the day and at night, and they are derived from different heme pools, which are presumably supplied by one or other of the two ferrochelatase isoforms FC1 and FC2. How heme synthesis is controlled, and whether this is dependent on specific subcompartmental localization of the proteins, should also be explored in the future.
5. If FLU is induced by PChlide sensing to inactivate GluTR in the dark, is GluTR also inactivated in a FLU-dependent manner under changing light conditions? If this is the case, it would again raise the question of how FLU-mediated GluTR inactivation is controlled (Hou *et al.*, 2021).
6. Does TBS require more rapidly responsive post-translational control mechanisms than other metabolic pathways of similar importance because of the photoreactive nature of some of the metabolic intermediates of TBS? The complex pathway, which synthesizes hazardous phototoxic intermediates, requires the concerted action of macromolecular complexes, which are strictly localized in subplastidial compartments. However, these are currently not well defined. Protein complexes made up of TBS enzymes have been reported by several research teams (Kauss *et al.*, 2012; Chidgey *et al.*, 2014; Knoppova *et al.*, 2014; Hey and Grimm, 2018, 2020) based on Blue Native gel electrophoresis, density-gradient centrifugation, and mass spectrometry. It is encouraging that these approaches provide evidence that TBS proteins form complexes with regulators and auxiliary factors. But it has become clear that the composition of these high-molecular-weight complexes is dynamically modified depending on the environmental conditions.
  As an example of the dynamic localization of TBS enzymes within chloroplasts, the regular shift of GluTR between the stroma and thylakoid membrane is correlated with ALA synthesis (Schmied *et al.*, 2018). The association of GluTR with the membrane in the dark and its release from the membrane in the light was detected in the wild type and becomes more pronounced in *flu* mutant or *FLU*-overexpressing lines (Kauss *et al.*, 2012; Schmied *et al.*, 2018; Hou *et al.*, 2019). Recent analyses revealed that, in Arabidopsis, POR, CURT1 (curvature Thy1), the MgCh subunit 1 CHLI, and CHLM are also localized to the grana margins (Wang *et al.*, 2016). Although there is increasing evidence to show that enzymes are assembled in macromolecular complexes for the tight regulation of metabolic flows, the organization of dynamically changing protein complexes for TBS in subcompartments of plastids is far from resolved. However, it is expected that future research will elucidate details of the subcompartmental organization of heme and Chl biosynthesis in plastids.
7. When the activities of enzymes in porphyrin, heme, and Chl synthesis are compromised, the increasing level of tetrapyrrole intermediates will generate photodynamic damage. Apart from the perception of inactive POR and apart from the heat-induced chaperone function on GluTR, CHLH, and GUN, do other protective and repair mechanisms exist for TBS enzymes when they are damaged and are necessarily replenished? Clearly, the protective mechanisms must have been developed in parallel to the establishment of TBS and also prior to the light-dependent PChlide reduction. However, apart from the FLU-dependent inactivation of GluTR and the heme-dependent degradation of GluTR, no protective mechanism has yet been described for TBS enzymes of the porphyrin and chlorin pathway that has the potential to protect against photodynamic damage. In this respect, TBS remains a fairly fragile metabolic pathway in the light, as photo-oxidation of vital metabolites can occur at any stage of TBS. To what extent reactive oxygen species-mediated or EXECUTER-dependent retrograde signaling may contribute to the protection of TBS enzymes will be examined in future.
8. Can the control mechanisms seen in chloroplasts be extrapolated to TBS in roots and flower organs? Almost all studies on plant TBS have been performed with leaf tissue. Therefore, all findings are based on TBS that is primarily devoted to Chl synthesis. Further studies should explore the regulation of TBS in roots and flowers, as it is likely to differ from that in aerial tissues, owing to the need for other quantities of other tetrapyrrole end-products.

Studies over the past two decades have joined up several loose ends in the network of TBS pathway control. But many gaps in our understanding of the control of TBS in plants remain to be filled.
